# Supplementary figures and images for: SARS-CoV-2 Nsp1 cooperates with initiation factors EIF1 and 1A to selectively enhance translation of viral RNA
Source: PLoS Pathog. 2024 Feb 9;20(2):e1011535. doi: 10.1371/journal.ppat.1011535 (PMC10903962; doi:10.1371/journal.ppat.1011535)

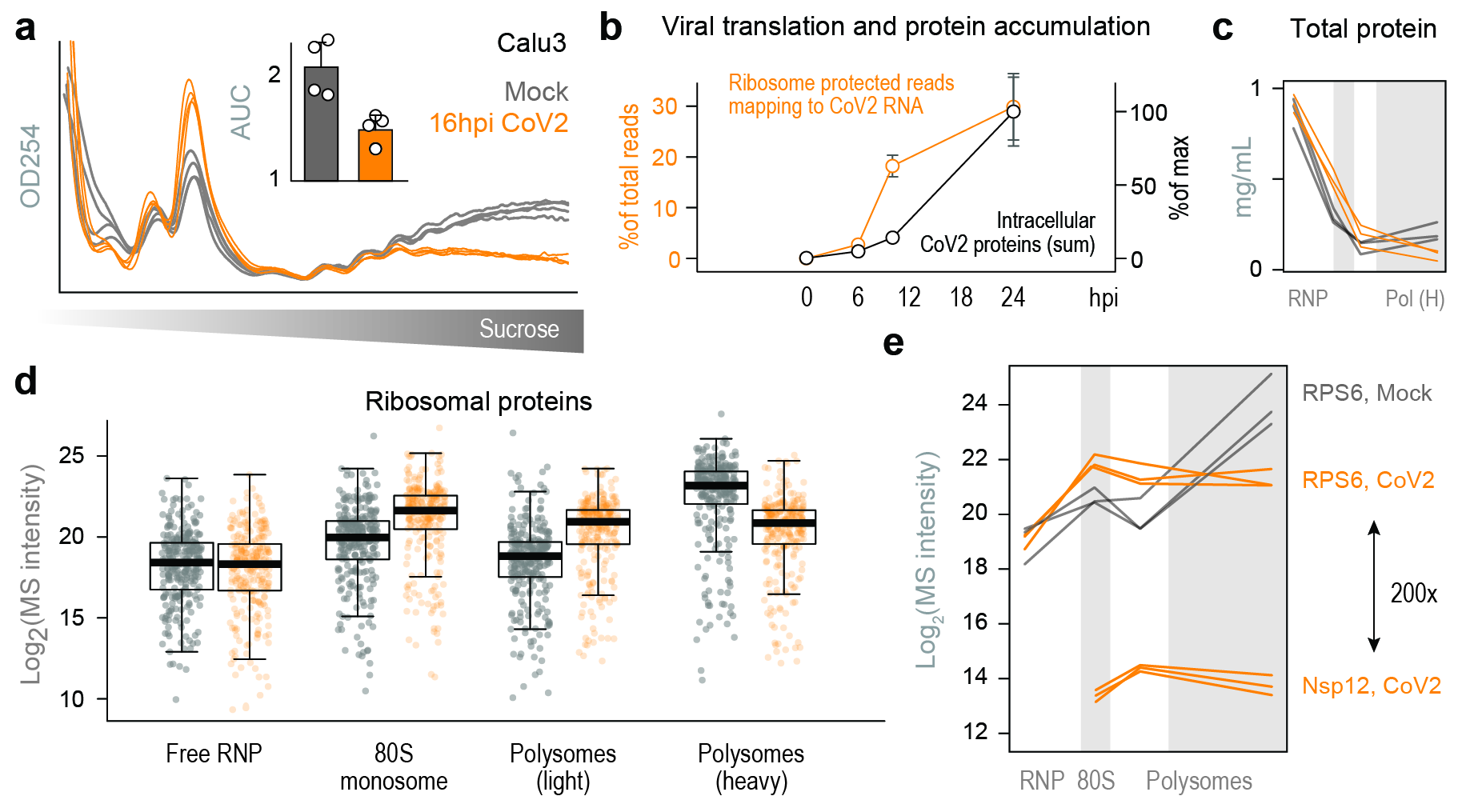

Supplement: S1 Fig — (a) Calu3 cells were infected with SARS-CoV-2 USA/WA1/2020 at MOI = 5, lysed, fixed with formaldehyde and fractionated on 10–50% sucrose gradients with continuous monitoring of rRNA absorbance. Each line reflects a single replicate, and bar graphs show the ratio of polysomes to sub-polysomes, calculated as the area under the curve (AUC) of relevant fractions. Shown are means±SD of 4 independent replicates. (b) Timecourse of CoV2 RNA translation and intracellular viral protein accumulation, from [10]. Shown are means±SD of 3 independent replicates. (c) Total protein extracted from pooled fractions of infected and uninfected cells, quantified by bicinchoninic acid (BCA) assays. Each line reflects a single replicate. (d) Boxplots of all cytosolic ribosomal proteins quantified by MS in each pooled fraction from all 3 replicates. (e) Line plots of RPS6 and CoV2 Nsp12 (RdRp) quantified by MS in each pooled fraction. Each line represents a single replicate. (TIF) [file ppat.1011535.s001.tif]

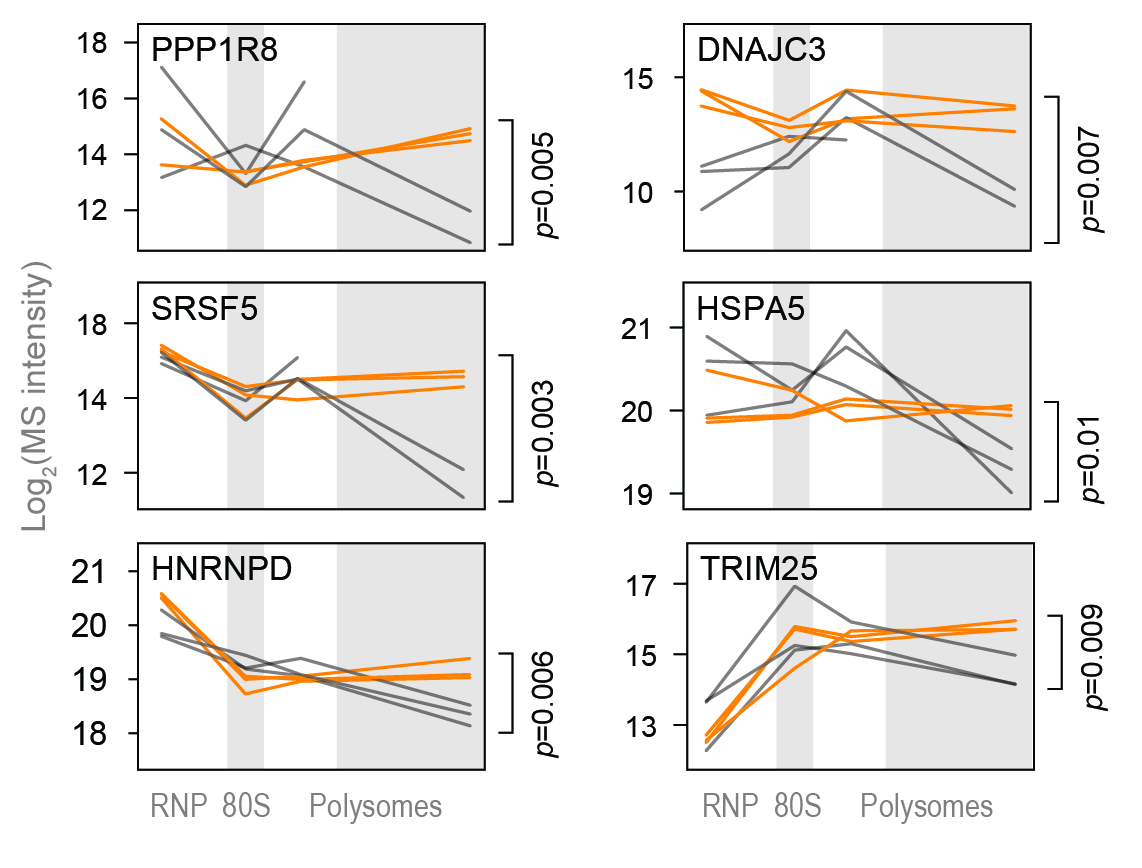

Supplement: S2 Fig — Line plots of individual ribosomal proteins quantified by MS in each pooled fraction. Each line represents a single replicate. P, two-tailed Student’s t-test p-value of differences in indicated protein abundance in heavy polysome fractions. (TIF) [file ppat.1011535.s002.tif]

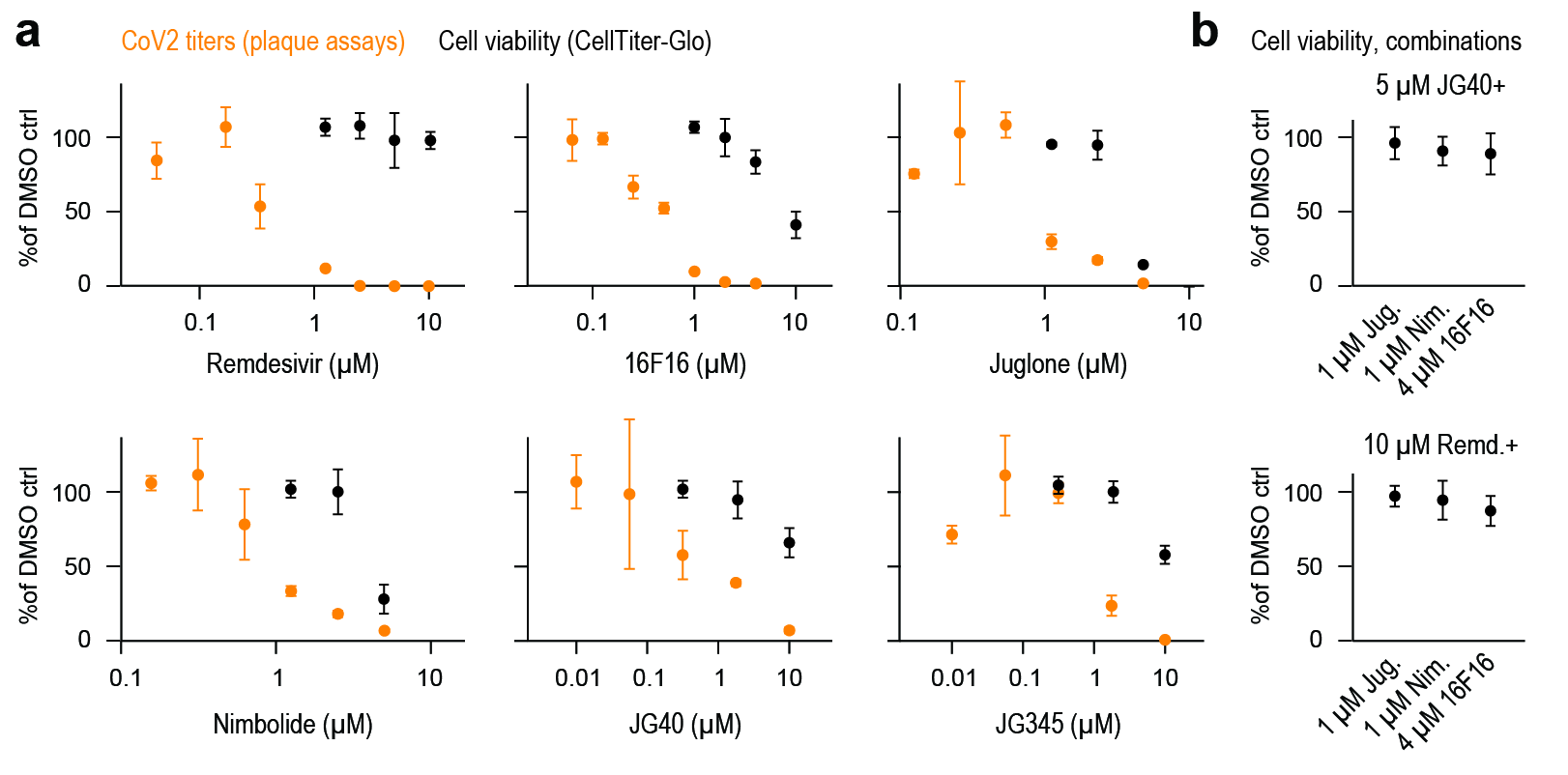

Supplement: S3 Fig — (a-b) Vero cells were infected with CoV2 at MOI = 0.5. Single drugs (a) or drug combinations (b) were added at the start of infection, and titers were determined by plaque assays at 16 hours post-infection. Toxicity was determined using CellTiter-Glo at 24h of drug treatment, in the absence of CoV2 infection. Shown are means±SD of 3 independent replicates, normalized to DMSO controls. (TIF) [file ppat.1011535.s003.tif]

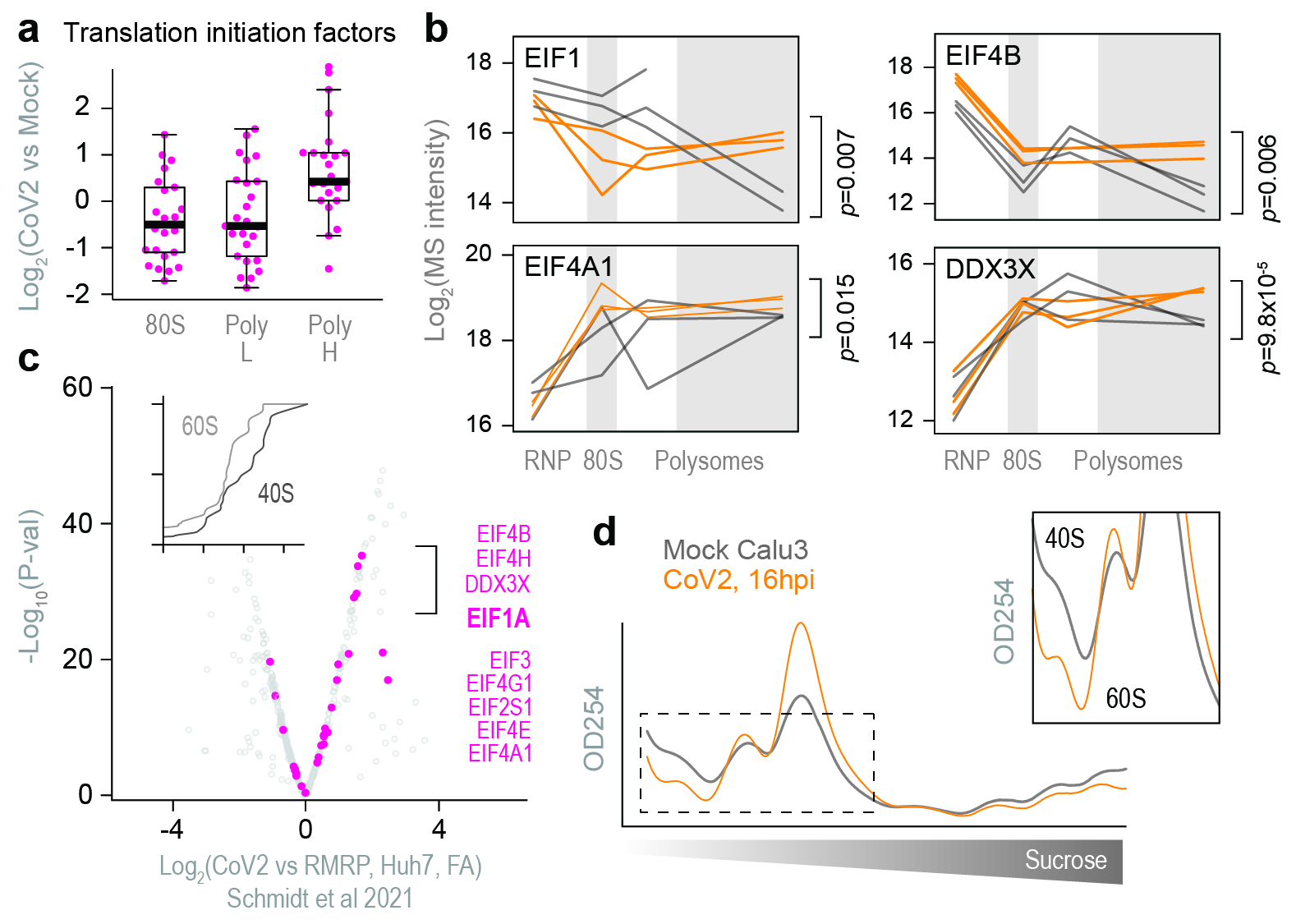

Supplement: S4 Fig — (a) Change in abundance of individual translation initiation factors upon CoV2 infection, in each fraction. (b) Line plots of individual translation factors quantified by MS in each fraction. Each line represents a single replicate. P, two-tailed Student’s t-test p-value. (c) Translation initiation factors are highly represented in the CoV2 RNA interactome during infection. Shown are pairwise comparisons of individual host protein abundance, quantified by MS, that specifically interact with either genomic or subgenomic CoV2 RNA. Inset, cumulative distribution plots of 40S and 60S ribosomal protein interaction with CoV2 RNA. (d) rRNA absorbance profiles showing lower abundance of free 40S subunits during CoV2 infection. Sum of four replicates. (TIF) [file ppat.1011535.s004.tif]

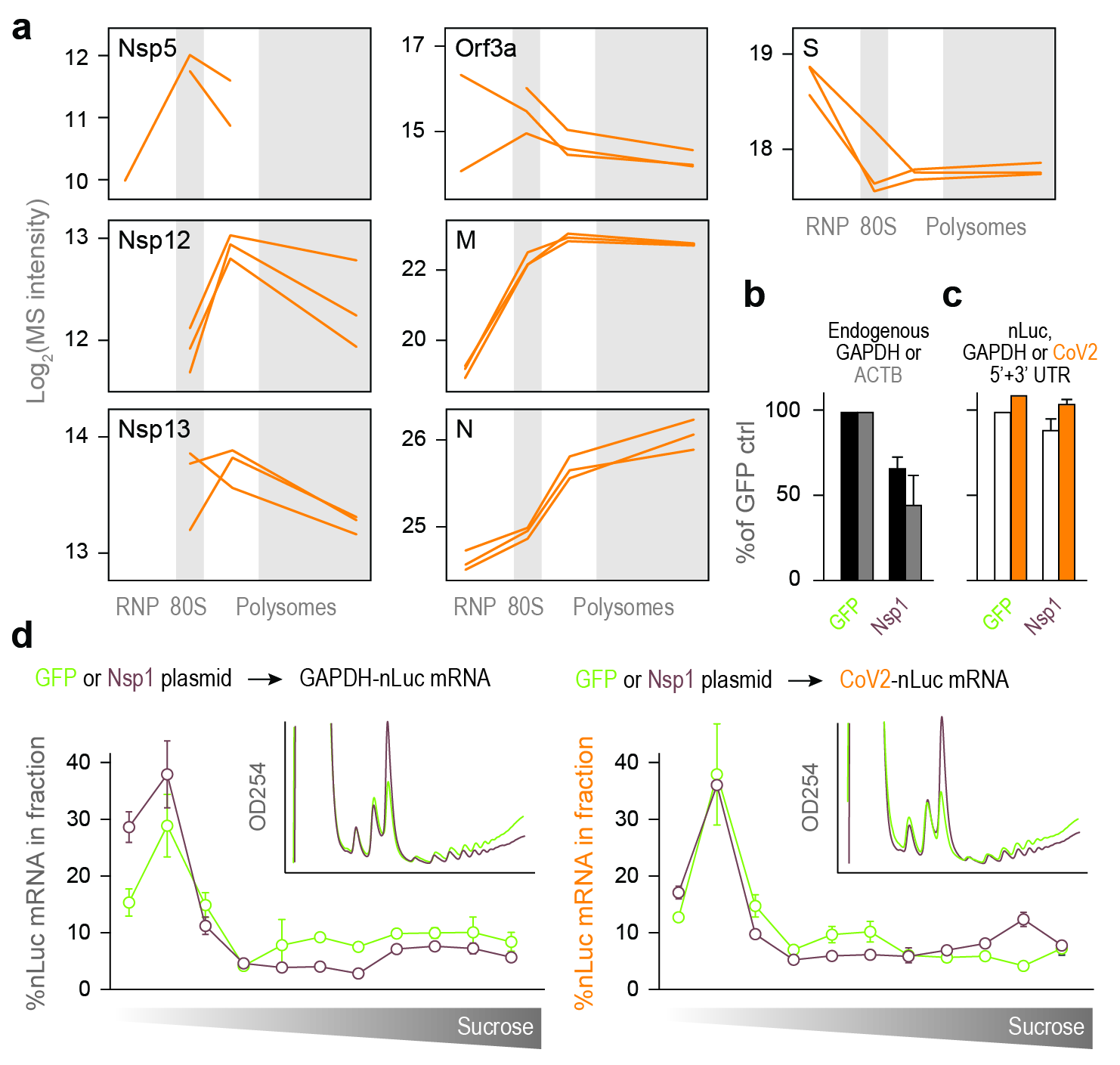

Supplement: S5 Fig — (a) Line plots of individual viral proteins quantified by MS in each fraction. Each line represents a single replicate. (b) Vero cells were transfected with either GFP or NSP1. At 24h, intracellular RNA was extracted and subjected to qPCR analysis using primers specific to the coding region of either GAPDH or ACTB, as well as 18S rRNA. Shown are means±SD of 2 independent replicates, normalized to rRNA levels. (c) Vero cells were transfected with either GFP or Nsp1. At 24h, cells were transfected again with GAPDH-nLuc or CoV2-nLuc mRNA. 4h post second transfection, intracellular RNA was extracted and subjected to qPCR analysis using primers specific to the coding region of nLuc. Shown are means±SD of 2 independent replicates, normalized to rRNA levels. (d) Cells transfected as above with either GFP or Nsp1 followed by GAPDH-nLuc or CoV2-nLuc mRNA were lysed and fractionated on 10–50% sucrose gradients with continuous monitoring of rRNA. The content of nLuc mRNA in each gradient fraction was determined by qPCR using primers specific to the coding region of nLuc. Gradient qPCR values were calculated as the relative proportion of nLuc mRNA in each fraction compared to the sum of all fractions. Shown are means±SD of 2 independent replicates. (TIF) [file ppat.1011535.s005.tif]

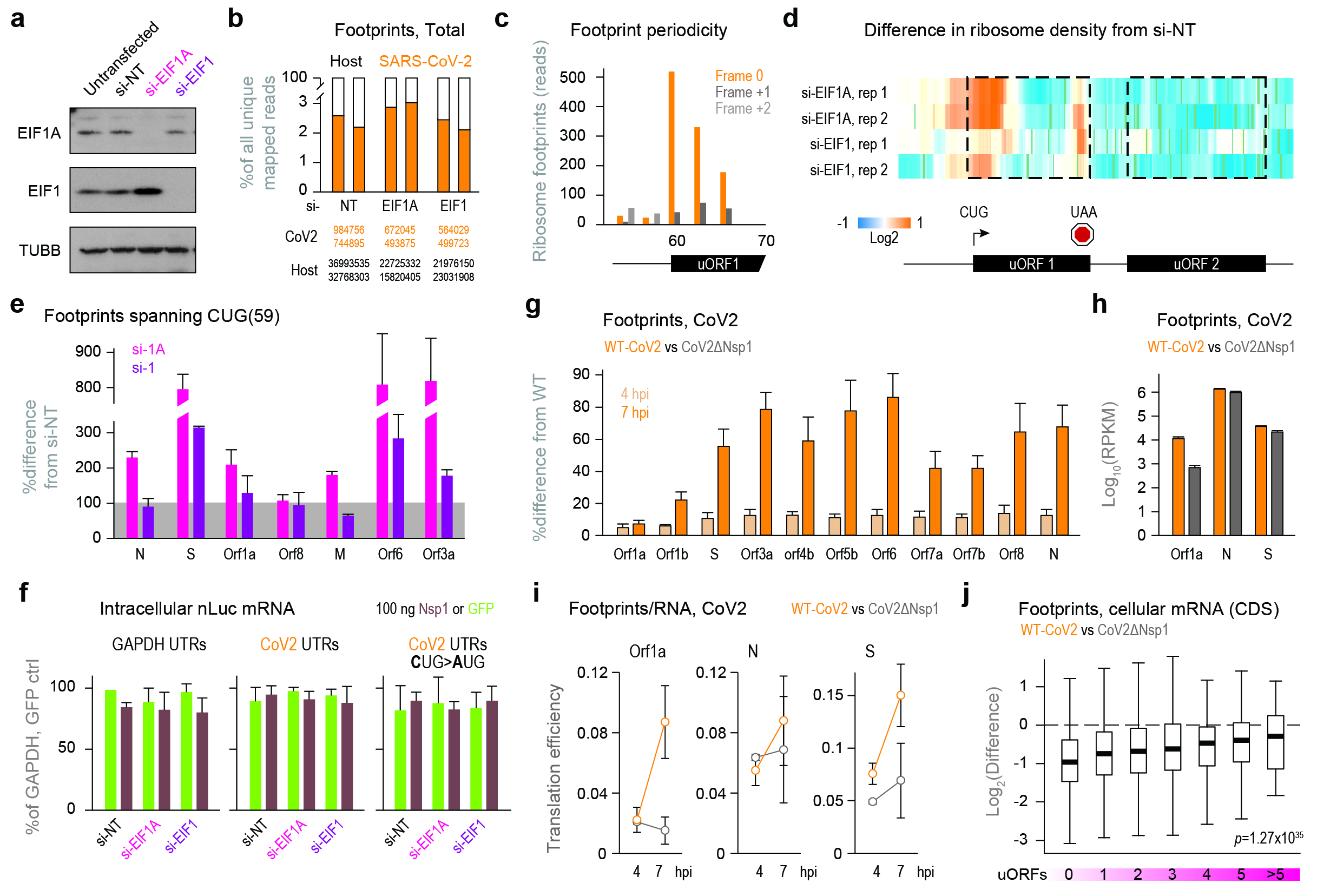

Supplement: S6 Fig — (a) Vero cells were transfected with siRNAs targeting initiation factors 1A and 1, compared to non-targeting (NT) controls. At 48h the same transfection was repeated. At 48h after the second transfection, cells were subjected to immunoblot analysis of whole cell lysates using the indicated antibodies. Shown are representative blots of 2 independent repeats. (b) Read mapping statistics for the ribosome profiling analyses in Fig 6B. Each bar represents a single biological replicate. (c) Periodicity analysis is consistent with translation of CoV2 gRNA uORF1 from CUG(59). P-site offsets were 13 nt from the 5’ end of each read. Shown is data from infection of control si-NT cells. (d) Heatmap showing log2 differences in ribosome occupancy on CoV2 gRNA 5’UTR between cells transfected with siRNAs against EIF1A and EIF1 as compared to non-targeting controls. (e) Footprints spanning CUG(59), assigned to gRNA (Orf1a) or individual sgRNA. (f) qPCR analysis of intracellular nLuc reporter mRNA in cells transfected with either GFP or Nsp1 followed by nLuc mRNA flanked by the indicated UTRs. Values were normalized to 18S rRNA. Shown are means±SD of 2 independent replicates. (g-i) Reanalysis of ribosome profiling datasets generated from Calu3 cells infected with either WT or Nsp1-dead CoV2. (g) Percent difference in footprints mapping to the indicated ORFs between WT-CoV2 and Nsp1-dead CoV2 at 4 hpi (light orange) and 7 hpi (dark orange). (h) Footprints mapping to Orf1a, N or S coding regions at 7 hpi. (i) Translation efficiencies of Orf1a, N and S, calculated as the ratio of ribosome protected reads to mRNA. Shown are means±SD of 2 independent replicates. (j) Boxplots showing the difference in translation of host mRNAs during infection with either WT or Nsp1-dead CoV2, binned by number of annotated uORFs. P, p-value of Mann-Whitney test comparing 0 and >5 uORFs. (TIF) [file ppat.1011535.s006.tif]
