# Supplementary material for: SARS-CoV-2 Nsp1 cooperates with initiation factors EIF1 and 1A to selectively enhance translation of viral RNA
Source: PLoS Pathog. 2024 Feb 9;20(2):e1011535. doi: 10.1371/journal.ppat.1011535 (PMC10903962; doi:10.1371/journal.ppat.1011535)
Supplement: Attachment — Submitted filename: PLoS Pathogen Rev1 rebuttal.docx [file ppat.1011535.s009.docx]

Dear editor,

We thank you for the opportunity to submit a revised version of our manuscript titled ***SARS-CoV-2 Nsp1 regulates translation start site fidelity to promote infection*** for further evaluation at PLoS Pathogens.

We thank the reviewers for their thoughtful comments, which helped us improve the overall quality of the manuscript and flesh out the details of our model. The revised manuscript now addresses all key questions raised during the review process, providing new ribosome profiling and reporter assay data as well as reanalysis of previously published datasets.

More specifically, we show that (a) ectopic expression of CoV2 Nsp1 destabilizes host mRNAs but not transfected, in-vitro transcribed reporter RNAs; (b) translation initiation factors EIF1A and EIF1, but not EIF4B, cooperate with Nsp1 to promote CoV2 infection by stimulating the bypass of an inhibitory upstream open reading frame (uORF) found in all viral RNAs; (c) mutating the start codon of this uORF from CUG to AUG strongly inhibits translation of the main ORF and prevents any Nsp1-mediated stimulation of translation; and (d) deletion of Nsp1 in the context of CoV2 infection decreases translation efficiency of viral genomic RNA as well as uORF-regulated host mRNAs.

Below is a detailed point-by-point response to the review comments.

**Part II – Major Issues: Key Experiments Required for Acceptance**

Reviewer #1: (No Response)

Reviewer #2: Major comments:

The authors present a model wherein a “functional interaction between Nsp1 and eIF4A1” reduces translation initiation on an upstream CUG start codon on the viral 5’ UTR, thereby selectively boosting the translation of viral transcripts. The model is intriguing, yet several controls are needed to strengthen this hypothesis.

Most importantly, previous studies have shown that an Nsp1-dependent translation boost is observed for reporter mRNAs carrying only SL1 of the viral UTR, yet this viral sequence does not contain the upstream CUG start codon. The authors should repeat the eIF1A depletion experiment with a reporter mRNAs carrying only the viral SL1 RNA to test if the functional interaction is indeed due to a repression of uORF translation. In addition, the authors should include a reporter mRNA in which the upstream CUG codon has been mutated.

Indeed, previous studies have found that CoV2 SL1 is sufficient for escaping the translation shutoff induced by Nsp1^1–4^. However, in the context of infection, all viral RNAs contain both SL1 and leader sequence, including CUG(59). This CUG codon was previously shown to drive efficient translation initiation of an upstream open reading frame (uORF1) in CoV2 genomic and subgenomic RNAs^5,6^. In fact, more ribosomes occupy this CUG as compared to downstream main AUG codons^5,6^. By mutating this CUG to AUG in reporter assays, we now show that translation of uORF1 inhibits that of the main ORF (Fig. 6g). Furthermore, we show that depletion of either EIF1 or 1A increases ribosome occupancy on the upstream CUG start codon as well as an in-frame UAA stop codon, and decreases occupancy on the main AUG and Orf1 coding region (Fig. 6c-e). These observations confirm that the 5’UTR of CoV2 RNAs harbors repressive elements, which are missing from all reporters that consist of SL1 alone. Therefore, SL1 is sufficient to confer efficient translation initiation in the presence of Nsp1 when placed immediately upstream to an AUG start codon. This creates an unusually short 5’UTR of about 30 nt (compared to a median of over 200 nt in humans)^7^, likely reflecting poor initiation context that could potentially benefit from the effects of Nsp1 on start site stringency.

In the revised version of the manuscript, we now show that, in the context of CoV2 full-length 5’UTR, replacing the upstream CUG with AUG prevents Nsp1-mediated stimulation of translation (Fig. 6g). Furthermore, CoV2 reporters escaped repression by Nsp1 at all concentrations, regardless of CUG(59), EIF1 or EIF1A (Fig. 6g), indicating that escape and stimulation may occur through distinct mechanisms. Finally, re-analysis of previously-published ribosome profiling datasets generated from cells infected with either WT or Nsp1-dead CoV2^8^ revealed that translation efficiency of Orf1 is hypersensitive to Nsp1 deletion (Fig. S6f-h). In conclusion, SL1 is essential for escaping translation repression by Nsp1, but it is the upstream CUG that mediates the stimulatory effects of Nsp1 on CoV2 gRNA translation, in a manner dependent on EIF1/1A.

According to the proposed model, Nsp1 overexpression should reduce translation initiation from the upstream CUG start codon. To strengthen this hypothesis, the authors should measure ribosome footprints on viral reporter RNA in control cells and cells overexpressing Nsp1 (ideally in the presence and absence of eIF1A).

We now show that intermediate (but not high) levels of ectopically expressed Nsp1 increase translation from CoV2 gRNA main AUG, in a manner dependent on the upstream CUG, EIF1A and EIF1 (Fig. 6g).

If Nsp1 recruits eIF1A to repress uORF translation on viral transcripts, the same mechanism would likely affect cellular transcripts. Previous studies showed the selective translation of cellular RNAs in the presence of Nsp1. Are these transcripts enriched for uORFs in the 5’ UTR?

To address this question, we reanalyzed published ribosome profiling datasets generated from cells infected with either WT or Nsp1-dead CoV2^8^. We find that translation of uORF-regulated host mRNAs is higher in the presence of functional Nsp1. Furthermore, this increase in translation of the main ORFs is proportional to the number of uORFs (Fig. S6i).

Additional comments:

- In Figure 5a the authors show that Nsp1 associates with polysomes, which is in contrast to previous studies that only found Nsp1 in 40S and 80S fractions of sucrose gradients. The authors should comment on this discrepancy. Is it likely due to higher sensitivity of the proteomic analysis, the formaldehyde crosslinking agents used in this study, or is it possible that the identified Nsp1 is part of nascent polypeptides synthesized on the ribosome?

Although some publications do suggest that Nsp1 may interact with polysomes^4,9^, others show that Nsp1 interaction with ribosomes is mutually exclusive with mRNA binding^10,11^. Whether or not Nsp1 can associate with polysomes through direct interactions with SL1 on nascent viral RNA is still contested^3,4,12,13^. As suggested by the reviewer, one potential explanation for such discrepancies is differences in the lower limit of detection of the assays used. Our analyses were performed using mass-spectrometry, which offers superior sensitivity as compared to traditional biochemical assays. Furthermore, samples were prepared using formaldehyde crosslinking, which could stabilize otherwise transient interactions.

In addition, the authors might reconsider the use of the word “significant” to describe the association of Nsp1 with polysome fractions, as no statistical test is shown to analyze the interaction.

We omitted the word “significant”. The new sentence now reads: “Still, Nsp1 was not entirely excluded from polysome fractions of infected cells (Fig. 5a).”

- In Figure 5d the authors use qPCR to measure the amount of reporter mRNAs associated with polysomes in the presence or absence of Nsp1, yet they fail show total cellular reporter mRNA levels. This is a crucial control, as it was previously shown that Nsp1 can induce mRNA cleavage and degradation, and that mRNAs harboring viral 5’UTR sequences are partially resistant to Nsp1-induced RNA cleavage. In will be important to know if the preferred association of viral reporter mRNAs with polysomes in the presence of Nsp1 is due to selective recruitment, or because viral mRNA is stabilized while cellular mRNA is degraded.

We have now added qPCR data to measure total transfected mRNA. Based on these analyses, included in Fig. S5b and S6j, we conclude that the effect of Nsp1 on translation initiation from CoV2 gRNA is distinct from its effects on mRNA cleavage or degradation. It was previously reported that in-vitro transcribed mRNAs are less sensitive to Nsp1-mediated degradation as compared to identical mRNA sequences generated by plasmid transfections^9^. Furthermore, potential destabilizing effects of Nsp1 were likely minimized by the short duration of the experiment, with intracellular mRNA and luminescence levels monitored 4 hours post-transfection.

- Figure 6d: The authors should include additional QC analysis of the ribosome profiling analysis, especially a graph showing the periodicity of the footprints in the ORF.

Read numbers as well as footprint periodicity plot are now included in Fig. S6b-c.

Reviewer #3: 1. In Figure 5c, the authors demonstrate that NSP1 enhances translation of the CoV2 gRNA. Given the destabilization of host mRNAs upon CoV2 infection or NSP1 expression, were there changes in the relative mRNA abundances in the presence of the viral proteins, which would confound interpretation of the translation data?

As mentioned above in response to a similar query from reviewer 2, qPCR data was added to Fig. S5b and S6j. Based on these analyses, included in Fig. S5b and S6j, we conclude that the effect of Nsp1 on translation initiation from CoV2 gRNA is distinct from its effects on mRNA cleavage or degradation. It was previously reported that in-vitro transcribed mRNAs are less sensitive to Nsp1-mediated degradation as compared to identical mRNA sequences generated by plasmid transfections^9^. Furthermore, potential destabilizing effects of Nsp1 were likely minimized by the short duration of the experiment, with intracellular mRNA and luminescence levels monitored 4 hours post-transfection.

2. In Figure 6, knockdown of eIF1A increased initiation at an upstream CUG start site in CoV2 gRNA relative to the canonical start site for ORF1a. The authors should examine how knockdown of other components of the 43S PIC impact initiation on the viral RNA. For example, upon knockdown of eIF1, eIF2 and eIF3, given the cooperativity between eIFs during formation of the 43S PIC. Such experiments are important because they would clarify whether the observed effects are specific to eIF1A or represent a more generalizable outcome of having defective initiation complexes.

In addition to EIF1A knockdown, we now present new data from ribosome profiling, reporter assays and immunoblot analyses of EIF1 knockdown, which was also enriched in heavy polysome fractions of CoV2-infected cells. Overall, EIF1 and EIF1A knockdown show similar effects (Fig. 6). These observations are consistent with the known function of both EIF1 and EIF1A in regulating translation start site selection^14–17^, particularly in the context of uORFs^18^. Furthermore, we now show that knocking down translation initiation factor EIF4B does not affect CoV2 titers (Fig. 6b), suggesting that the effects observed upon EIF1/1A depletion are not a generalized outcome of interfering with translation initiation.

3. Does knockdown of eIF1A reduce the enrichment of other eIFs in the heavy polysome fraction upon CoV2 infection (i.e., how does eIF1A knockdown affect the enrichments reported in Fig. 4a)? Also, is the apparent lack of eIF2 enrichment in the heavy polysome fractions upon CoV2 infection (Figure 4a) a technical limitation or potentially relevant?

We thank the reviewer for these intriguing suggestions. Addressing them would require additional laborious experiments and, in our opinion, will help expand our understanding but not strengthen the main arguments of the current work. To reflect these queries, we added the following statement to the discussion: “Future work will need to determine how EIF1/1A depletion remodels polysome interactions during CoV2 infection, and whether additional factors contribute to initiation fidelity on CoV2 RNAs.”

4. In Figure 6g, the authors show that the NSP1-mediated enhancement of gRNA translation by NSP1 required eIF1A. This is a very important figure in their manuscript, and it is missing a key component. The authors again should compare and/or report the mRNA levels of the reporter in each condition. Is it a translation effect or an RNA stability effect?

As discussed above in response to other queries, qPCR data are now included in Fig. S5b and S6j. Given the lack of significant differences in intracellular levels of the transfected reporters, we conclude that the observed differences in luminescence are not due to destabilization of reporter mRNA.

5. The authors cite one paper related to eIF1A function during initiation, which happens to support the authors’ observations. However, eIF1A has a multifaceted role and there has been substantial prior effort to understand its function both in vitro and in cells, in both mammals and yeast. The authors should place the findings they report here in the context of prior studies (e.g., foundational work by the Pestova, Hellen, Fraser, Hinnebusch, Dever, & Lorsch groups).

We thank the reviewer for the opportunity to provide a more thorough review of current literature pertaining to the role of EIF1A and EIF1 in translation initiation. Additional references are now included in both the Results and Discussion sections.

**Part III – Minor Issues: Editorial and Data Presentation Modifications**

Reviewer #1: 1. The paper appears to be written for a shorter format journal, and given that PPATH allows more text space, the significance of the findings would land with more impact if the authors fleshed out their central questions and experimental logic/interpretations more thoroughly. The figure legends also lack sufficient detail for easy understanding. For example, color coded labeling should be described in the legend.

The manuscript text was edited to provide a more thorough description and discussion of the observations and conclusions.

2. Fig. 4f: please clarify how the composition (e.g., length, GC content) of the GAPDH UTR sequences compare to those of CoV2. In the spectrum of host mRNA translation efficiencies, where does GAPDH lie (is it average? highly efficient?).

The 5’UTR of GAPDH is shorter than that of CoV2 gRNA (103 vs 265 nt) and has a higher GC content (61 vs 44). GAPDH is a highly abundant mRNA (99^th^ percentile) with relatively high translation efficiency (90^th^ percentile) in HEK293 cells^19^. GAPDH translation efficiency decreases upon stress, suggesting it does not possess any elements that stimulate translation under stress^19^. GAPDH UTRs have been used as controls for both viral and cellular translation regulatory elements^20–24^.

The following was added to the first mention of GAPDH UTRs:

“To determine whether this reflects a lower rate of translation initiation on CoV2 RNA, we generated in-vitro transcribed, capped and polyadenylated nano-luciferase (nLuc) mRNAs flanked by 5’ and 3’ UTRs of either GAPDH or CoV2 gRNA. GAPDH UTRs were chosen as reference based on their prior use as controls for efficient constitutive translation, including in the context of CoV2 infection.”

3. The significance of the reduced level of nsp1 in polysomes relative to other ORF1a/b proteins is not clear (Fig. 5a). Why are Nsp2/3/4/8/10 polysome associated? If they are there simply because they are in the process of being translated (rather than for a regulatory reason), is nsp1 relatively absent from polysomes because it is released more rapidly from the polyprotein than the others?

As discussed in the above response to reviewer 2, some publications suggest that Nsp1 may interact with polysomes^4,9^ while others show that Nsp1 interaction with ribosomes is mutually exclusive with mRNA binding^10,11^ and therefore should not occur on polysomes. Whether or not Nsp1 can associate with polysomes through interactions with SL1 on nascent viral RNA is still contested^3,4,12,13^. One potential explanation for such discrepancies is differences in the lower limit of detection of the assays used. Our analyses were performed using mass-spectrometry, which offers superior sensitivity as compared to traditional biochemical assays. Furthermore, samples were prepared using formaldehyde crosslinking, which could stabilize otherwise transient interactions.

4. A key finding is that depletion of EIF1A prevents nsp1 from boosting the translation efficiency of the CoV2 leader-containing RNA (Fig6f-g). I assume this mechanism should only work on viral genomic RNA, not subgenomic RNA (indicated in Fig.6F). Yet, other reports have shown that reporters containing the CoV2L derived from sgRNA escape nsp1 repression in transfection experiments. More discussion is warranted on this point, e.g., is it a difference between escaping repression and translational enhancement? Additionally, it would be valuable to include a version of the luc reporter where the upstream CUG is mutated in the Fig.6g assay, to confirm the extent to which the role of EIF1A involves readthrough of that codon.

Please see our detailed response to reviewer 2, above (pages 1-2). In brief, we now show that both EIF1A and EIF1 facilitate the Nsp1-mediated stimulation of gRNA translation and that replacing the upstream CUG with AUG prevents such stimulation independently of the levels of EIF1/1A (Fig. 6g).

Reviewer #2: - Extended Figure 1b – legend is missing. Please describe what is shown with the orange and grey line. Please use different color coding, since grey is used for mock-infected cells throughout the manuscript.

Extended Fig. 1b was rearranged for clarity.

- Figure 3: label for panel “d” is missing.

We are unsure what the reviewer is referencing here, given that a 3d panel was never included in the original submission.

- Figure 6g: The authors should show absolute luciferase activity, not just normalized data, to demonstrate how much the depletion of eIF1A changes the overall translation of all reporter constructs.

Data analysis and visualization were revised as suggested (Fig. 6g).

Reviewer #3: 1. Extended data Fig 1e is a bit unexpected. Why are there more RPs present in light polysomes relative to mock, despite identical polysome curves? The data do not appear to support this statement: “The abundance of ribosomal proteins in each pooled fraction, as quantified by MS (Fig. 1f-g and Extended Data Fig. 1d), was consistent with the observed rRNA absorbance profiles (Fig. 1d).”

We thank the reviewer for pointing this out. Our labeling and description of the different pooled fractions in Fig. 1f was inaccurate. The revised figure now shows that the light polysome fractions contain more ribosomes based on both rRNA absorbance profiles and mass-spec quantification.

2. In the sucrose gradients used for the polysome fractionation experiments, where does the CoV2 gRNA migrate? It was hinted at early in the text, but does it migrate into the heavy polysome fractions? It could be helpful to analyze migration of in vitro transcribed gRNA on the gradient to determine this. This is relevant mechanistically because it might help distinguish whether a viral gRNA contains a single, paused 40S initiation complex, or whether the gRNA contains 80S ribosomes and an initiation 40S complex.

We thank the reviewer for this suggestion. Unfortunately, such experiments are complicated by the need to crosslink infected samples for biosafety reasons, forming stable RNA-protein complexes that impede reverse transcription and therefore qPCR analysis. Furthermore, generating the full-length gRNA by in-vitro transcription is complicated by its length of ~30,000 nt RNA. Nevertheless, Fig. 5d and S5c show the migration of in-vitro transcribed nanoluciferase mRNA flanked by the 5’ and 3’ UTRs of CoV2 gRNA.

3. In figure 4f, does removing the uORF yield similar translation as GAPDH? In other words, is it the uORF or the complex secondary structure that limits translation of the gRNA? How does gRNA translation compare to sgRNA translation?

Ribosomes were shown to initiate translation from 5 distinct sites on the 5’UTR of CoV2 gRNA^6^. Previous work has also shown that mutating the upstream CUG to CCG affects different CoV2 sgRNAs in different ways, for example decreasing translation from Spike UTR but increasing translation from Orf7a UTR^5^. In the current work, we show that knockdown of either EIF1A or EIF1 alters the relative usage of each translation initiation site during infection (Fig. 6d, S6d), as well as the occupancy on an in-frame stop codon (Fig. S6d), suggesting complex and interdependent dynamics of initiation. We also show that mutating the upstream CUG to AUG to impose translation of uORF1 dramatically reduces translation from the main AUG, independently of EIF1/1A levels, and prevents the Nsp1-mediated stimulation of translation (Fig 6g). Furthermore, we show that the different effects of EIF1/1A depletion on translation of different CoV2 RNAs are reflected at the level of the encoded proteins (Fig. 6f).

4. As far as we are aware, all structures of the NSP1-bound ribosome do not contain mRNA. So, this statement is not supported by the published data: “This suggested that Nsp1 may preferentially interact with or stabilize 48S pre-initiation complexes with specific composition or function, as supported by cryo-EM studies of Nsp1-bound ribosomes.”

This comment was deleted.

As discussed in the above response to reviewers 1 and 2, some publications suggest that Nsp1 may interact with polysomes^4,9^ while others show that Nsp1 interaction with ribosomes is mutually exclusive with mRNA binding^10,11^ and therefore should not occur on polysomes. Whether or not Nsp1 can associate with polysomes through interactions with the nascent viral RNA is still contested^3,4,12,13^. One potential explanation for such discrepancies is differences in the lower limit of detection of the assays used. Our analyses were performed using mass-spectrometry, which offers superior sensitivity as compared to traditional biochemical assays. Furthermore, samples were prepared using formaldehyde crosslinking, which could stabilize otherwise transient interactions.

5. “It is tempting to speculate that Nsp1 stabilizes eIF1A binding or otherwise affects its interactions with the ribosome decoding center, although additional work is needed to elucidate the specifics of the mechanism.” This is unlikely given this preprint (Universal features of Nsp1-mediated translational shutdown by coronaviruses), which shows that SARS-CoV-2 NSP1 does not impact eIF1A association or dissociation from 40S subunits, albeit in the absence of other eIFs.

This statement was deleted, and the above-referenced paper was cited.

6. In figure 6g, what happens if you remove the CUG(60) on CoV2? Does NSP1 lose all stimulatory effects on translation relative to GFP? How is translation then impacted after transfecting with Si-1A?

As discussed above in response to point #4, Fig. 6g now shows that mutating the upstream CUG to AUG in the context of the full-length CoV2 gRNA 5’UTR dramatically reduces translation from the main AUG, independently of EIF1/1A levels, and prevents the Nsp1-mediated stimulation of gRNA translation.

7. From figure 6a and figure 4a, it appears RNA helicases eIF4A1 and DDX3 both associate to NSP1 and are recruited to heavy polysome fractions during CoV2 infection. Complex RNA secondary structure has been shown to promote upstream CUG translation initiation, suggesting helicases could be important for translation of downstream ORFs. It would be interesting to see if knockdown of those two helicases also decreases NSP1-mediated stimulation of translation on CoV2 gRNA.

We thank the reviewer for this suggestion. Indeed, others have shown that DDX3 and related RNA helicases interact with CoV2 UTRs and play pro-viral roles during infection^25–27^. Additional studies are required to determine whether these RNA helicases also contribute to accurate start site selection on CoV2 RNA. However, such intriguing experiments fall beyond the scope of the current work.

The following statement was added to the discussion: “Many RNA helicases are also known to bind CoV2 RNA and modulate infection. Future work will need to determine how EIF1/1A depletion remodels polysome interactions during CoV2 infection, and whether additional factors contribute to initiation fidelity on CoV2 RNAs.”

References

1.	Vora, S. M. *et al.* Targeting stem-loop 1 of the SARS-CoV-2 5′ UTR to suppress viral translation and Nsp1 evasion. *Proc. Natl. Acad. Sci.* **119**, (2022).

2.	Bujanic, L. *et al.* The key features of SARS-CoV-2 leader and NSP1 required for viral escape of NSP1-mediated repression. *RNA* **28**, 766–779 (2022).

3.	Banerjee, A. K. *et al.* SARS-CoV-2 Disrupts Splicing, Translation, and Protein Trafficking to Suppress Host Defenses. *Cell* **183**, 1325-1339.e21 (2020).

4.	Tidu, A. *et al.* The viral protein NSP1 acts as a ribosome gatekeeper for shutting down host translation and fostering SARS-CoV-2 translation. *RNA* **27**, 253–264 (2021).

5.	Kim, D. *et al.* A high-resolution temporal atlas of the SARS-CoV-2 translatome and transcriptome. *Nat. Commun.* **12**, 5120 (2021).

6.	Finkel, Y. *et al.* The coding capacity of SARS-CoV-2. *Nature* **589**, 125–130 (2021).

7.	Leppek, K., Das, R. & Barna, M. Functional 5′ UTR mRNA structures in eukaryotic translation regulation and how to find them. *Nat. Rev. Mol. Cell Biol.* **19**, 158–174 (2018).

8.	Fisher, T. *et al.* Parsing the role of NSP1 in SARS-CoV-2 infection. *Cell Rep.* **39**, 110954 (2022).

9.	Slobodin, B. *et al.* Cap-independent translation and a precisely located RNA sequence enable SARS-CoV-2 to control host translation and escape anti-viral response. *Nucleic Acids Res.* **50**, 8080–8092 (2022).

10.	Schubert, K. *et al.* SARS-CoV-2 Nsp1 binds the ribosomal mRNA channel to inhibit translation. *Nat. Struct. Mol. Biol.* **27**, 959–966 (2020).

11.	Thoms, M. *et al.* Structural basis for translational shutdown and immune evasion by the Nsp1 protein of SARS-CoV-2. *Science (80-. ).* **369**, 1249–1255 (2020).

12.	Vankadari, N., Jeyasankar, N. N. & Lopes, W. J. Structure of the SARS-CoV-2 Nsp1/5′-Untranslated Region Complex and Implications for Potential Therapeutic Targets, a Vaccine, and Virulence. *J. Phys. Chem. Lett.* **11**, 9659–9668 (2020).

13.	Mendez, A. S. *et al.* The N-terminal domain of SARS-CoV-2 nsp1 plays key roles in suppression of cellular gene expression and preservation of viral gene expression. *Cell Rep.* **37**, 109841 (2021).

14.	Passmore, L. A. *et al.* The Eukaryotic Translation Initiation Factors eIF1 and eIF1A Induce an Open Conformation of the 40S Ribosome. *Mol. Cell* **26**, 41–50 (2007).

15.	Martin-Marcos, P. *et al.* eIF1A residues implicated in cancer stabilize translation preinitiation complexes and favor suboptimal initiation sites in yeast. *Elife* **6**, (2017).

16.	Pestova, T. V., Borukhov, S. I. & Hellen, C. U. T. Eukaryotic ribosomes require initiation factors 1 and 1A to locate initiation codons. *Nature* **394**, 854–859 (1998).

17.	Saini, A. K., Nanda, J. S., Lorsch, J. R. & Hinnebusch, A. G. Regulatory elements in eIF1A control the fidelity of start codon selection by modulating tRNA i Met binding to the ribosome. *Genes Dev.* **24**, 97–110 (2010).

18.	Zhou, F., Zhang, H., Kulkarni, S. D., Lorsch, J. R. & Hinnebusch, A. G. eIF1 discriminates against suboptimal initiation sites to prevent excessive uORF translation genome-wide. *RNA* **26**, 419–438 (2020).

19.	Sidrauski, C., McGeachy, A. M., Ingolia, N. T. & Walter, P. The small molecule ISRIB reverses the effects of eIF2α phosphorylation on translation and stress granule assembly. *Elife* **4**, (2015).

20.	Lapointe, C. P. *et al.* Dynamic competition between SARS-CoV-2 NSP1 and mRNA on the human ribosome inhibits translation initiation. *Proc. Natl. Acad. Sci.* **118**, (2021).

21.	Lee, S., Park, D., Lim, C., Kim, J.-I. & Min, K.-T. mtIF3 is locally translated in axons and regulates mitochondrial translation for axonal growth. *BMC Biol.* **20**, 12 (2022).

22.	Therizols, G. *et al.* Alteration of ribosome function upon 5-fluorouracil treatment favors cancer cell drug-tolerance. *Nat. Commun.* **13**, 173 (2022).

23.	Erales, J. *et al.* Evidence for rRNA 2′-O-methylation plasticity: Control of intrinsic translational capabilities of human ribosomes. *Proc. Natl. Acad. Sci.* **114**, 12934–12939 (2017).

24.	Takahashi, K. *et al.* Critical Roles of Translation Initiation and RNA Uridylation in Endogenous Retroviral Expression and Neural Differentiation in Pluripotent Stem Cells. *Cell Rep.* **31**, 107715 (2020).

25.	Vesuna, F. *et al.* RK-33, a small molecule inhibitor of host RNA helicase DDX3, suppresses multiple variants of SARS-CoV-2. *Front. Microbiol.* **13**, (2022).

26.	Ariumi, Y. Host Cellular RNA Helicases Regulate SARS-CoV-2 Infection. *J. Virol.* **96**, (2022).

27.	Ciccosanti, F. *et al.* Proteomic analysis identifies the RNA helicase DDX3X as a host target against SARS-CoV-2 infection. *Antiviral Res.* **190**, 105064 (2021).
