# Supplementary material for: SARS-CoV-2 Nsp1 cooperates with initiation factors EIF1 and 1A to selectively enhance translation of viral RNA
Source: PLoS Pathog. 2024 Feb 9;20(2):e1011535. doi: 10.1371/journal.ppat.1011535 (PMC10903962; doi:10.1371/journal.ppat.1011535)
Supplement: Attachment — Submitted filename: PLoS Pathogen Rev2 rebuttal_RA JF RA.docx [file ppat.1011535.s010.docx]

There is one significant technical concern raised. Specifically, Reviewer #2 is concerned about whether there is protection from mRNA degradation of the reporter in the presence of NSP1 and whether the transfection methods used might impact the conclusions. We would appreciate if you could address this either by providing additional data or by explaining why this is not a concern.

In addition, Reviewer #2 makes an important suggestion about rewording the claim about the role of NSP1 in start codon selection to state that eIF1A appears to regulate the Nsp1-dependent translation boost. We would encourage you to include this or similar language in the manuscript to note this remaining uncertainty.

Please prepare and submit your revised manuscript within 30 days. If you anticipate any delay, please let us know the expected resubmission date by replying to this email.

When you are ready to resubmit, please upload the following:

[1] A letter containing a detailed list of your responses to all review comments, and a description of the changes you have made in the manuscript.

Please note while forming your response, if your article is accepted, you may have the opportunity to make the peer review history publicly available. The record will include editor decision letters (with reviews) and your responses to reviewer comments. If eligible, we will contact you to opt in or out

[2] Two versions of the revised manuscript: one with either highlights or tracked changes denoting where the text has been changed; the other a clean version (uploaded as the manuscript file).

**Part I - Summary**

**Reviewer #1**: The authors have nicely addressed all my prior concerns.

**Reviewer #2**: The authors have substantially revised and significantly improved the manuscript. In particular, the analysis of ribosome footprinting data of Nsp1-deficient and WT viruses strongly strengths the presented model.

However, the revision failed to sufficiently address 2 of my previous concerns (outlined below, related to the role of Nsp1 in regulating RNA stability and the direct connection between Nsp1 and uORF translation). In the current form, the presented data is insufficient to support the model that Nsp1 regulates eIF1A-dependent start codon selection on viral RNAs. An additional round of minor revisions is therefore suggested, before the manuscript is ready for publication.

**Part II – Major Issues: Key Experiments Required for Acceptance**

**Reviewer #1**: (No Response)

**Reviewer #2**: 1. One of the major concerns was that total reporter mRNA levels were not measured in the previous version of this manuscript. It was therefore impossible to determine whether the viral 5'UTR protected from Nsp1-dependent RNA degradation or Nsp1-dependent translation repression. In the revised manuscript, the authors include total RNA quantification, and conclude that luciferase reporter mRNA levels are unchanged. However, the authors use lipofectamine for direct mRNA transfection, which makes a quantification of *accessible* cytoplasmic mRNA impossible. It is very likely that a majority of transfected mRNA is still contained in lipid vesicles within the cell, and therefore protected from degradation. A different transfection method (e.g. electroporation) or DNA transfection is needed to draw conclusions about RNA stability. Without additional experiments, the authors would need to weaken their statement to convey that, based on the presented data, they cannot exclude a primary effect of the 5'UTR on RNA stability.

We thank Reviewer #2 for this comment. Indeed, lipid-based transfection of in-vitro synthesized mRNA results in partial retention of mRNA in lipid vesicles, rendering it inaccessible to cytoplasmic processing by e.g. ribosomes and nucleases. Still, this method is commonly used for introducing translation-competent mRNAs into cells, including delivery of CoV2 Spike mRNA in COVID19 vaccines. Importantly, we fail to see how these concerns affect the interpretation of our results, as outlined below.

Our work focuses on previously undescribed aspects of CoV2 translation regulation. Nowhere in the manuscript do we argue that CoV2 RNA stability is not an important determinant of viral protein expression, nor that Nsp1 does not destabilize host mRNAs. However, we do provide results from multiple orthogonal assays that directly probe the **efficiency of translation from CoV2 5’UTR** with and without Nsp1. As previously reported, we show that Nsp1 expression increases the total level of nLuc protein produced from CoV2 5’UTR, without affecting **the intracellular levels of the transfected mRNA** (Fig. 4f, 5c, S5b-c, 6g, S6j). These analyses do not address the caveat pointed out by the reviewer but indicate that, under these conditions, RNA stability does not contribute to the stimulatory effects of NSP1 on CoV2 5’UTR translation. We also show, using sucrose gradient coupled to qPCR, that Nsp1 **shifts CoV2 5’UTR-nLuc from 80S** **to** **heavy polysome fractions** (Fig. 5d, S5d). These experiments conclusively demonstrate a role for Nsp1 in translation initiation. In addition, we present an analysis of ribosome profiling datasets from cells **infected** with either WT or *Nsp1-null* CoV2. These analyses show that loss of Nsp1 reduces the efficiency of translation from CoV2 5UTR by a factor of 4 (Fig. S6f-h).

Given that (a) transfected mRNAs harboring CoV2 SL1 are known to be protected from Nsp1-induced degradation; (b) our CoV2 reporters all contain SL1; and (c) the intracellular levels of these reporter mRNAs are unaffected by Nsp1 expression (Fig. 5c, S5b-c, 6g, S6j), the above observations cannot be explained by differences in RNA stability. Therefore, our conclusion that Nsp1 enhances translation initiation from CoV2 5’UTR is the most compelling explanation that fits all of our data, and is **unlikely** to change with other transfection methods.

Since, as previously reported, we also find that endogenous cellular mRNAs are degraded by Nsp1 (Fig. S5b), we revised the text to acknowledge that the partial resistance of transfected GAPDH 5’UTR mRNA to Nsp1 expression (Fig. S5c) may reflect partial protection by endocytic vesicles. This concern, which only applies to our control GAPDH reporters, is now reflected in the **following new statement** (new text in bold):

“Expression of Nsp1 reduced the levels of endogenous GAPDH and ACTB (Fig. S5b) but had no effect on the levels of transfected nLuc mRNAs (Fig. S5c). This is likely due to the lower sensitivity of in-vitro transcribed RNAs to Nsp1-mediated degradation^27^ and the short duration of the experiment (4 h post RNA transfection). **However, it is also possible that a fraction of transfected mRNAs is retained in endocytic vesicles, which may protect them from Nsp1-induced degradation and potentially confound measurements of intracellular mRNA stability, thus obscuring the destabilizing effects of Nsp1 on host mRNA**.

2. While the authors present a vast amount of data supporting the hypothesis that viral uORF translation is regulated by eIF1A (and eIF1), the link between Nsp1 and uORF translation is still weak. The only direct evidence comes from Fig. 6g, which shows that eIF1A-depletion reduces the Nsp1-dependent boost in viral reporter mRNA translation. While this experiment shows a link between eIF1A and Nsp1, it does not show that this link is dependent on the upstream CUG start codon. The CUG-->AUG mutant is insufficient, because it almost completely abolishes all translation. If the authors want to make the statement that Nsp1 regulates start codon usage via eIF1A, they should repeat the experiment of Fig. 6g with a reporter that does not have a upstream start codon at all. This is particularly relevant as the Nsp1-dependent translation boost has been shown for reporter mRNAs that only carry the viral SL1, and no upstream start codon. Alternatively or additionally, changes in ribosomal foot prints on upstream start codons in the presence and absence of Nsp1 (similar to Figure 6c) could be shown to strengthen the link between Nsp1 and start codon selection. In the absence of either of these important experiments, the authors should should not conclude that Nsp1 regulates start codon selection, they can merely state that eIF1A appears to regulate the Nsp1-dependent translation boost.

We appreciate the comments of Reviewer #2 agreeing with our conclusion that eIF1 and 1A regulate viral uORF translation. As discussed in the manuscript, CoV2 5’UTR harbors five AUG and non-AUG start codons that, based on ribosome profiling data, are used to initiate translation during infection (Fig. 6a). As expected, these start sites are interdependent, because increased usage of an upstream site decreases that of downstream sites (Fig. 6d). Therefore, to test the role of Nsp1 in uORF bypassing on CoV2 5’UTR we would need to carefully delete each upstream start site individually and in combination with others, generating 16 different reporters, and test those under 6 different conditions (+/- Nsp1, eIF1A KD, eIF1 KD). Though intriguing, such experiments would be hard to interpret and fall beyond the scope of the current work.

Nevertheless, we do show that eIF1A—and to a lesser extent, eIF1—promotes uORF bypassing on CoV2 5’UTR by controlling start site selection, resulting in increased translation of the main ORF (Fig. 6d, S6d-e). Furthermore, we show that eIF1A—and to a lesser extent, eIF1—is important for the stimulatory effects of Nsp1 on main ORF translation (Fig. 6g). We also show that mutating the upstream CUG to AUG enforces upstream initiation and restricts, though not completely abrogates, the stimulatory effects of Nsp1. Importantly, and as reported before, CoV2 5’UTR escapes repression by Nsp1 across all conditions tested, suggesting multiple mechanisms of action. Supporting this hypothesis is the complex non-linear relationship between Nsp1 levels and translation stimulation (Fig. 6g). We offer additional support for this conclusion by providing an analysis of previously published ribosome profiling data that shows that Nsp1 increases main ORF translation on both viral and host transcripts (Fig. S6h-i).

Together, these observations clearly implicate eIF1/1A and Nsp1 in regulating the balance of translation initiation between upstream and downstream ORFs. Although the precise nature of interactions between Nsp1 and eIF1/1A should be probed further, we believe that the observations provided are enough to conclude that Nsp1 regulates start site selection, at least indirectly.

Still, to address this concern from Reviewer #2, we revised the title of the manuscript to “*SARS-CoV-2 Nsp1 cooperates with initiation factors EIF1 and 1A to selectively enhance translation of viral RNA*” and added the following statement at the end of the manuscript: “**Although it remains unclear whether Nsp1 can regulate translation start site selection independently of eIF1/1A, this interdependency may present potential new targets for antiviral therapies.**”

**Part III – Minor Issues**: Editorial and Data Presentation Modifications

**Reviewer #1**: (No Response)

**Reviewer #2**: The statement "A conserved stem-loop (SL1) found at the 5’ end of all viral RNAs (Fig. 6a) displaces Nsp1 from the ribosome" should be revised. There is currently no direct evidence that SL1 directly displaces Nsp1 from the ribosome, only that SL1 containing RNAs escape the Nsp1-dependent repression.

Although some published work suggests physical interactions between SL1 and Nsp1 (e.g. Vankadari et al 2020; Mendez et al 2021), we acknowledge that this remains controversial. Therefore, we revised the above statement:

“**A conserved stem-loop (SL1) found at the 5’ end of all viral RNAs (Fig. 6a) protects from Nsp1-induced repression^7,48^, though it remains unclear whether such protective effects are mediated by direct interactions that displace Nsp1 from ribosomes.**”
